# Effective Privacy Protection Strategies for Pregnancy and Gestation Information From Electronic Medical Records: Retrospective Study in a National Health Care Data Network in China

**DOI:** 10.2196/46455

**Published:** 2024-08-20

**Authors:** Chao Liu, Yuanshi Jiao, Licong Su, Wenna Liu, Haiping Zhang, Sheng Nie, Mengchun Gong

**Affiliations:** 1 Digital Health China Technologies Co, Ltd Beijing China; 2 Department of Nephrology Nanfang Hospital Southern Medical University Guangzhou China; 3 School of Biomedical Engineering, Guangdong Medical University Zhanjiang China

**Keywords:** pregnancy, electronic medical record, privacy protection, risk stratification, rule-based

## Abstract

**Background:**

Pregnancy and gestation information is routinely recorded in electronic medical record (EMR) systems across China in various data sets. The combination of data on the number of pregnancies and gestations can imply occurrences of abortions and other pregnancy-related issues, which is important for clinical decision-making and personal privacy protection. However, the distribution of this information inside EMR is variable due to inconsistent IT structures across different EMR systems. A large-scale quantitative evaluation of the potential exposure of this sensitive information has not been previously performed, ensuring the protection of personal information is a priority, as emphasized in Chinese laws and regulations.

**Objective:**

This study aims to perform the first nationwide quantitative analysis of the identification sites and exposure frequency of sensitive pregnancy and gestation information. The goal is to propose strategies for effective information extraction and privacy protection related to women’s health.

**Methods:**

This study was conducted in a national health care data network. Rule-based protocols for extracting pregnancy and gestation information were developed by a committee of experts. A total of 6 different sub–data sets of EMRs were used as schemas for data analysis and strategy proposal. The identification sites and frequencies of identification in different sub–data sets were calculated. Manual quality inspections of the extraction process were performed by 2 independent groups of reviewers on 1000 randomly selected records. Based on these statistics, strategies for effective information extraction and privacy protection were proposed.

**Results:**

The data network covered hospitalized patients from 19 hospitals in 10 provinces of China, encompassing 15,245,055 patients over an 11-year period (January 1, 2010-December 12, 2020). Among women aged 14-50 years, 70% were randomly selected from each hospital, resulting in a total of 1,110,053 patients. Of these, 688,268 female patients with sensitive reproductive information were identified. The frequencies of identification were variable, with the marriage history in admission medical records being the most frequent at 63.24%. Notably, more than 50% of female patients were identified with pregnancy and gestation history in nursing records, which is not generally considered a sub–data set rich in reproductive information. During the manual curation and review process, 1000 cases were randomly selected, and the precision and recall rates of the information extraction method both exceeded 99.5%. The privacy-protection strategies were designed with clear technical directions.

**Conclusions:**

Significant amounts of critical information related to women’s health are recorded in Chinese routine EMR systems and are distributed in various parts of the records with different frequencies. This requires a comprehensive protocol for extracting and protecting the information, which has been demonstrated to be technically feasible. Implementing a data-based strategy will enhance the protection of women’s privacy and improve the accessibility of health care services.

## Introduction

Medical information is generally considered to be highly sensitive for individuals, and any breach of privacy can cause direct or indirect harm to patients [1]. For female patients, pregnancy and gestation information is not only highly private but also implies the incidence of abortion, which is extremely controversial in terms of the rights and responsibilities of women in some jurisdictions [2-4]. Evidence suggests that the leakage of such information can negatively impact the attitudes of patients’ social environment and even health care providers [4,5].

The worldwide implementation of electronic medical records (EMRs) has significantly improved patient care by making health information readily accessible to a wide range of data producers. From 2007 to 2018, the average adoption rates of EMR increased from 18.6% to 85.3% [6]. This rapid growth has led to the processing and storage of various categories of patient information, including demographics, medications, laboratory tests, and diagnostic records, thereby establishing EMR as a valuable resource for large-scale data analysis of real-world data. However, the unprecedented use of EMR posed new challenges for protecting patient information effectively and preventing the unnecessary exposure of sensitive data during real-world evidence (RWE) research. Consequently, there is growing attention to the legal and technical research on extracting pregnancy and gestation information and the relevant privacy protection strategies [7,8].

On March 26, 2021, the Binhai Procuratorate accepted and examined a case of infringement of citizens’ personal information. Staff responsible for preventive health care at a town town-central health center in Binhai County, Jiangsu province, took advantage of their positions to illegally obtain the family contact information and home addresses of pregnant women and newborns, totaling 25,124 items. This information was then resold through digital platforms, resulting in an illegal profit of US $4566 and subjecting pregnant women to telephone harassment. In response to this phenomenon, starting in 2022, local authorities began conducting annual comprehensive inspection of the supervision of fertility information and specifically informed the procuratorial organs of the inspection results [9]. New laws and regulations have also come out, such as the “Guangdong Province Maternal and Child Health Care Management Regulations” began to implement, which came into effect on June 1, 2023. These regulations emphasize the confidentiality of personal information and privacy in maternal and child health care services and related supervision and management [10].

According to the “Technical Specifications for Hospital Information Platforms Based on EMR” issued by the National Health Commission of China in 2014, different health institutions in the country share a similar EMR framework comprising several sub–data sets including diagnostic information, medical advice, laboratory test results, examination information, and surgical records [11]. However, issues of discontinuity and incompleteness in EMR writing pose significant challenges in multicenter data integration [12]. Traditional information extraction and privacy protection strategies during RWE research and clinical data transfer have primarily focused on fixed sub–data sets, such as marriage and childbearing history, and direct data entities like the number of pregnancies in patients’ EMRs. These approaches, known as fixed site recognition strategies, lead to biased patient inclusion and flawed data masking in RWE research. For pregnancy and gestation information, testing results and procedures can indicate pregnancy status and gestation incidence without explicit descriptions in diagnostic sheets. For instance, a surgical history of pregnancy termination can imply suction aspiration abortion, while pregnancy history can be inferred from clinical test results such as human chorionic gonadotropin (HCG) levels exceeding 10 ng/L or 25 IU/L [13,14].

This study aims to propose protocols for the accurate and automatic extraction of pregnancy and gestation information from Chinese EMRs at the highest possible level of precision. Such information is crucial for patient inclusion and cohort identification in RWE studies to improve pregnancy outcomes [15,16]. Additionally, privacy protection strategies will be developed to maximize the masking of pregnancy data and identify the risk of privacy leakage for different sub–data sets within EMRs. To the best of our knowledge, this study is the first to identify the frequency of privacy information in Chinese EMRs. Then, the related risks can be considered when using patients’ EMRs for RWE research.

## Methods

### Data Source

This retrospective study uses the Chinese Renal Disease Data System (CRDS) database, a comprehensive national EMR database. The CRDS includes data from 19 tertiary referral hospitals across 10 provinces, representing the 5 geographical regions of China (North, Central, East, South, and Southwestern). Each hospital’s database covers the EMRs of all patients who visited from the start of 2010 to the end of 2020. The patient’s EMRs were not specially selected. Complete EMRs from each hospital were transferred to the central database located at Nanfang Hospital of Southern Medical University in Guangzhou. In this study, the total number of patients in the database is 15,245,055. All analyzed hospitalization records were structured based on the CRDS data model [17].

### Sample Patient Inclusion

In this study, female patients aged 14-50 years from January 1, 2010, to December 31, 2020, were selected from the CRDS database. The statistical time here was the patient’s last visit information (including all the previous visit history), and 70% (n=1,110,053) were randomly selected for statistical analysis.

### Extraction of Pregnancy and Gestation Information From Chinese EMRs

Following a preliminary investigation of Chinese EMRs, and incorporating expert guidance, teaching materials, guidelines, and literature, the research team developed the Extraction Protocol of Pregnancy and Gestation Information (EPPGI). This protocol was refined through repeated sorting, adjustment, and verification, considering the writing characteristics of various hospital medical records. Traditional methods typically extract patient data using diagnosis codes from the diagnostic sheets of Chinese EMRs. However, we first developed identification rules for test and exam results, covering patients with positive HCG results in different units of measurement and pregnancy tests.

Given the diversity and complexity of the medical coding system in Chinese EMRs, we used regular expressions (regex) to retrieve pregnancy and gestation information across entire EMRs rather than relying solely on diagnosis codes in specific sub–data sets. The adopted regex extended beyond diagnoses to include surgical procedures, chief complaints related to pregnancy status, and gestation histories. Besides, regex for medications related to inducing labor or miscarriage was used to assist in identifying pregnancy information. Other regex, including description of fetus and exclusion rules, was also applied. All regex search patterns were the product of expert meetings and discussions. The detailed rules and regex of EPPGI are listed in Multimedia Appendix 1.

To implement this approach, we used R software (version 4.2.2; R Core Team) to extract females with reproductive activities (FRA) information from the checklist using regular expressions. In the following example, “final_medtech” represents the checklist, and “TECHNOLOGY_RESULT” is the field containing the check result in the checklist.



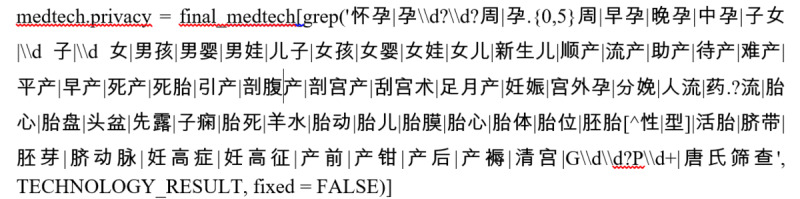



The rules of regex allowed us to describe the proportion of patients with a pregnancy history across different sub–data sets of EMRs and the frequency of pregnancy and gestation information. After removing duplicate patients from different sub–data sets, we retrieved data on pregnant women in the selected EMRs using EPPGI.

### Privacy Protection Strategies

Based on the statistics of the located information, we proposed privacy protection strategies to avoid unnecessary and unintentional exposure of pregnancy and gestation information in real-world data analytics. Due to the different writing styles in medical records, insufficient desensitization may not fully cover sensitive patient information, while excessive desensitization may obscure other relevant information. First, EPPGI was used to identify keywords of sensitive reproductive information (SRI), such as “助产|难产|平产|早产|死产|死胎” (“midwifery|dystocia| normal birth|preterm birth|stillbirth|stillbirth”). With expert guidance, we finally chose to replace 15 characters before and after these keywords with asterisks (*) to desensitize sensitive information related to pregnancy and childbirth, thereby protecting patient privacy. This approach minimizes the possibility of inferring patients’ SRI from EMRs.

In cases where the use of maternity-related information is unavoidable, the frequency of patient identification and privacy information was used to estimate the risk of unnecessary privacy exposure methodically. We also used diagnosis and marital history as criteria to locate maternity information and compared these results across 6 large sub–data sets of EMRs. A total of 2 independent reviewers (WL and HZ) inspected both methods to ensure accuracy and reliability.

### Manual Curation and Verification

Afterward, included cases were randomly selected and manually reviewed by 2 independent groups of reviewers (CL, YJ, LS, WL, HZ, SN, and MG) to test the precision and recall of the data extraction. For the EPPGI, 1000 female cases were randomly assigned to 2 external experts (Aixin Guo and Wenna Liu) to manually extract SRI. The manually extracted results were then compared with the EPPGI results to evaluate the precision and recall rate, as defined below. We also compared the precision and recall rates of the EPPGI with those obtained using only maternal and diagnostic history.

**Figure 1:**
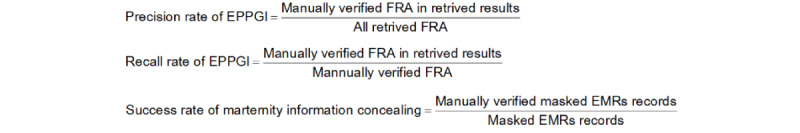
Formulas for precision, recall, and success rate. Precision is the ratio of correctly predicted positive observations to the total predicted positives, focusing on the accuracy of the positive predictions. Recall is the ratio of correctly predicted positive observations to all the actual positives, focusing on the ability to capture all actual positive cases. EMR: electronic medical record; EPPGI: Extraction Protocol of Pregnancy and Gestation Information; FRA: females with reproductive activities.

Additionally, the reviewers attempted to identify FRA in privacy-concealed data sets to test the success rate of the privacy protection strategies, as defined in Figure 1.

### Ethical Considerations

This study was approved by the Medical Ethics Committee of Nanfang Hospital, Southern Medical University (approval NFEC-2019-213), which waived the requirement for patient-informed consent due to the retrospective nature of the study. This study was also approved by the China Office of Human Genetic Resources for Data Preservation Application (approval 2021-BC0037). This study complied with the Declaration of Helsinki and the STROBE (Strengthening the Reporting of Observational Studies in Epidemiology) statement.

## Results

To the best of our knowledge, this study is the first to identify the frequency of privacy information in Chinese EMRs.

### General Information of EMRs

**Figure 2:**
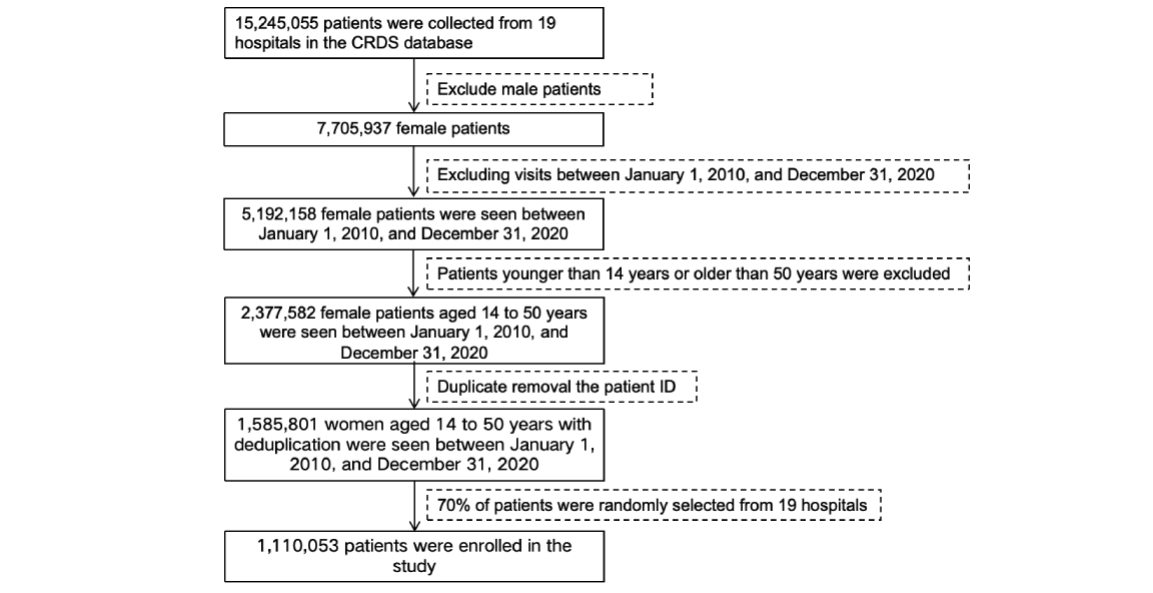
Admittance flowchart. The initial database had 15,245,055 patients. After excluding male patients, specific years, teenagers, and older adults, removing duplicate patient records, and applying a 70% entry ratio, the final sample consisted of 1,110,053 observations. CRDS: chinese renal disease data system.

**Figure 3:**
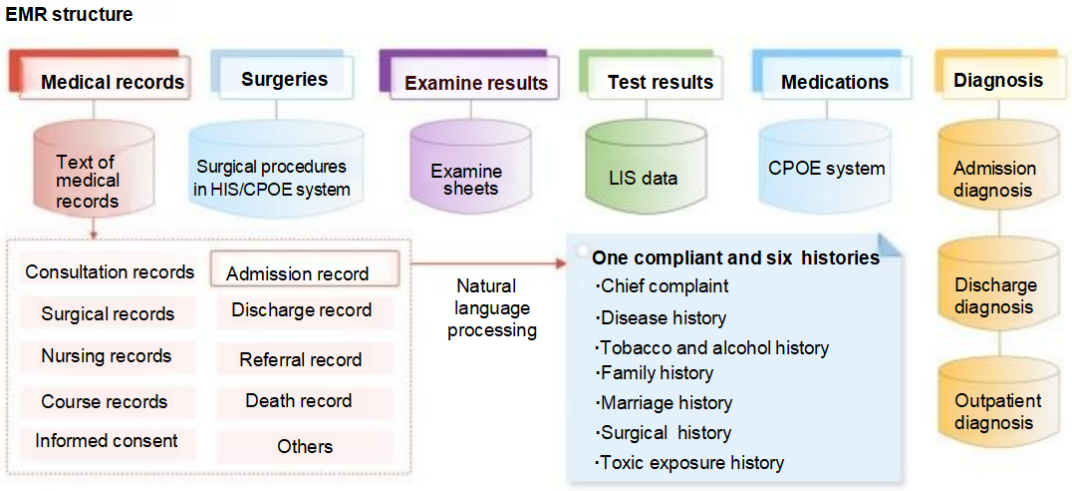
Structure of Chinese EMRs. The EMRs consist of medical records, surgery, examination results, test results, medication, and diagnosis. CPOE: computerized physician order entry; CRDS: chinese renal disease data system; EMR: electronic medical record; HIS: hospital information system; LIS: laboratory information system; NLP: natural language processing.

All patient data were extracted from the CRDS database, a real-world database that includes records from 19 hospitals. Based on the inclusion criteria (female patients aged 14-50 years from January 1, 2010, to December 31, 2020) and a 70% entry ratio, a total of 1,110,053 patients were selected as the EMR sample. It is worth noting that removing duplicates reduced the sample size from 2,377,582 to 1,585,801, which is due to multiple diagnostic records for individual patients. The admittance flowchart is shown in Figure 2, and detailed information is displayed in Table 1. According to Chinese national specifications for standard EMR structure, EMRs consist of similar sub–data sets with minor differences in nomenclature including doctor’s orders, diagnostic tables, test sheets, examination sheets, surgical sheets, and medical record texts. The medical record texts are further divided into 10 parts: course records, admission records, discharge records, referral records, consultation records, nursing records, death records, surgical notes, informed consent forms, and others. In CRDS, the admission record texts have been preprocessed using natural language processing for allergic history, chief complaint, disease history, tobacco and alcohol history, family history, marriage history, surgical history, and toxic exposure history. The general structure of Chinese EMRs is demonstrated in Figure 3*.*

**Table 1:** General information on EMRsa from 19 hospitals.

Hospital number	City and area	Total bed numbers	FRA^b^ (n=688,268)	Total patients (n=1,110,053)
1	Guangzhou, Southern	2225	57,837	102,483
2	Beijing, Northern	1650	30,410	36,757
3	Jinan, Northern	4000	79,294	94,339
4	Hangzhou, Eastern	3200	30,950	70,523
5	Hangzhou, Eastern	2400	67,086	82,977
6	Guangzhou, Southern	3000	53,355	82,654
7	Shenzhen, Southern	2000	41,352	48,269
8	Nanjing, Eastern	2499	33,709	51,919
9	Shanghai, Eastern	800	499	743
10	Chengdu, Southwestern	1000	21,803	78,843
11	Hefei, Eastern	3138	61,044	103,203
12	Wuhan, Central	5613	2555	4858
13	Maoming, Southern	2500	64,299	81,673
14	Guangzhou, Southern	2247	22,406	54,663
15	Huizhou, Southern	2156	22,756	23,181
16	Guiyang, Southwestern	2000	166	6138
17	Foshan, Southern	2200	63,336	125,085
18	Guangzhou, Southern	3000	6358	20,100
19	Guangzhou, Southern	1000	29,053	41,645

^a^EMR: electronic medical record.

^b^FRA: females with reproductive activities.

### Number of FRA

After the initial investigation, we applied the EPPGI to a sample of 1,110,053 female patients of childbearing age. This analysis covered 6 different categories of EMRs, with each sub–data set and its components processed separately. Table 2 presents the total number of patients, the identified number of FRA, and their corresponding proportions.

**Table 2:** Patient identified by EPPGIa. From left to right, the columns display the total number of patients, the number of identified FRAb, and their corresponding proportions.

EMR^c^ sub–data sets		Patient number (per person)	Maternal patient number		Percentage (%)
Order		955,140	146,555		15.34
Diagnosis from the frontage		1,073,167	312,008		29.07
Laboratory report		903,987	93,386		10.33
Examine result		852,143	172,735		20.27
Prescription of surgical procedures in HIS^d^/CPOE^e^ system		767,693	157,027		20.45
**Medical records**					
	Total	588,963	393,550	66.82	
	Course records	207,575	95,012	45.77	
	Discharge records	330,909	112,927	34.13	
	Referral records	9699	2682	27.65	
	Consultation records	38,728	12,639	32.64	
	Nursing records	192,080	112,465	58.55	
	Death records	953	105	11.02	
	Surgical notes	134,889	43,280	32.09	
	Informed consent	238,014	102,386	43.02	
	Others	411,637	138,892	33.74	
**Admission records (NLP^f^)**					
	Total	376,176	317,962	84.52	
	Allergic history	446,360	0	0.00	
	Chief complaint	13,925	55	0.39	
	Disease history	446,390	11,312	2.53	
	Tobacco and alcohol history	490,028	0	0.00	
	Family history	464,516	6	0.00	
	Marriage history	467,184	295,436	63.24	
	Surgical history	316,282	74,118	23.43	
	Toxic exposure history	504,022	4	0.00	

^a^EPPGI: Extraction Protocol of Pregnancy and Gestation Information.

^b^FRA: females with reproductive activities.

^c^EMR: electronic medical record.

^d^HIS: hospital information system.

^e^CPOE: computerized physician order entry.

^f^NLP: natural language processing.

The number of pregnancies identified solely by diagnosis was 312,008, accounting for 29.07% of the patients in the diagnostic sub–data set. The number of patients who were identified only by their marital and childbearing history was as high as 295,436, accounting for 26.61% of the total study population. The number of pregnancies identified by diagnosis and marital and childbearing history was 521,132, accounting for 46.95% of the total study population. If on the basis of diagnosis and marital history, the identification of diagnosis, examination, and other contents are added, the number of maternity information can be identified as 688,268, accounting for 62% of the total study population.

In the text of medical records, 393,550 patients with SRI were identified, accounting for 66.82% of 588,963 records. Due to the presence of childbearing history, which constitutes the leading source of SRI, over 80% (n=317,962) of female patients in admission records were identified as FRA by EPPGI. Besides, 58.55% (n=112,465) of female patients were identified in nursing history, making it the second highest proportion of FRA.

Based on these results, EPPGI effectively extracts FRA from every sub–data set within Chinese EMRs.

### Frequency of Recognition

A single patient can generate multiple encounter records in the EMR system per visit. Therefore, individual EMRs were divided into separate records based on visits, reflecting the actual EMR storage in RWE studies. Table 3 presents the frequency of pregnancy information identification across different sub–data sets of Chinese EMRs. Similar to the results from per-patient records, SRI can be widely identified in each sub–data set of EMRs. SRI is primarily concentrated on diagnosis records, surgical records, and medical records text. In diagnosis records, SRI could be extracted from 15.06% of 15,497,063 records. In surgical records, 11.49% of SRI could be extracted from 1,604,579 records. The text of medical records showed the highest frequency of SRI identification, with an overall recognition rate of 29.92%. Additionally, it is noteworthy that more than 80% of admission records contained SRI.

**Table 3:** Frequency of pregnancy information identification. From left to right, the columns display the total number of records, the frequency of identified FRA^a^, and their corresponding proportions.

EMR^b^ sub–data sets			Record number (per visit)		Maternal record number		Percentage (%)
Order			93,182,790		384,699		0.41
Diagnosis from the frontage			15,497,063		2,334,160		15.06
Laboratory report			102,509,232		285,245		0.28
Examine result			6,790,300		549,078		8.09
Prescription of surgical procedures in HIS^c^/CPOE^d^ system			1,604,579		184,335		11.49
**Medical records**							
	Total	8,473,462		2,534,940		29.92	
	Course records	2,132,926		527,915		24.75	
	Discharge records	532,790		151,352		28.41	
	Referral records	25,171		6737		26.76	
	Consultation records	151,564		43,695		28.83	
	Nursing records	965,042		268,586		27.83	
	Death records	2250		166		7.38	
	Surgical notes	482,578		106,656		22.10	
	Informed consent	1,226,875		326,284		26.59	
	Others	2,377,080		637,807		26.83	
**Admission records (NLP** ^e^ **)**							
	Total	577,186		465,742		80.69	
	Allergic history	1,183,577		0		0.00	
	Chief complaint	45,987		59		0.13	
	Disease history	4,166,715		15,057		0.36	
	Tobacco and alcohol history	858,066		0		0.00	
	Family history	2,076,062		6		0.00	
	Marriage history	708,544		444,559		62.74	
	Surgical history	553,774		106,846		19.29	
	Toxic exposure history	3,954,196		4		0.00	

^a^FRA: females with reproductive activities.

^b^EMR: electronic medical record.

^c^HIS: hospital information system.

^d^CPOE: computerized physician order entry.

^e^NLP: natural language processing.

### Precision and Recall Rate

During the manual curation and certification process, 1000 complete EMRs were randomly selected from the sample patients and reviewed by 2 independent medical experts (Aixin Guo and Wenna Liu) to determine maternal status. The precision and recall rates of the EPPGI were 100% and 99.68%, respectively. When only diagnosis history and marital history were used for identification, the accuracy rate remained 100%, but the recall rate dropped to 73.35%. For details, see Tables 4 and 5, where “0” represents patients without FRA information and “1” represents patients with FRA information.

**Table 4:** Confusion matrix of EPPGI^a^ method.

Prediction	Reference	
	0	1
0	377	2
1	0	621

^a^EPPGI: Extraction Protocol of Pregnancy and Gestation Information.

**Table 5:** Confusion matrix of the method using only diagnosis and marital history

Prediction	Reference	
	0	1
0	377	166
1	0	457

We also conducted analyses by time and region, as shown in Tables 6 and 7. In these tables, “quality inspection” refers to patients assessed by 2 expert manual reviews for quality control to determine the presence of labor process information (from different hospital sources); “EPPGI” refers to patients assessed using EPPGI for maternity information; and “diagnosis history and marital history” refers to patients assessed using diagnosis and marital history for fertility information. In these tables, 0 represents “no maternity information” and 1 represents “there is maternity information.” The results indicated that similar to the overall comparison, the identification of maternal information using the EPPGI method was superior to using diagnosis and marital history alone. By examining the results across different hospitals and time periods, our method proved to be universally applicable across various years and regions.

**Table 6:** FRA^a^ identification results of different privacy methods in different hospitals (1 being “there is maternity information” and 0 being “no maternity information”).

Hospital number	Quality inspection		EPPGI^b^		Diagnosis history and marital history	
	0	1	0	1	0	1
						
1	40	49	40	49	57	32
10	57	18	57	18	62	13
11	40	54	40	54	45	49
12	3	4	3	4	3	4
13	15	64	16	63	42	37
14	29	16	29	16	29	16
15	0	17	0	17	3	14
16	2	1	2	1	3	0
17	48	55	48	55	71	32
18	17	7	17	7	17	7
19	6	31	6	31	9	28
2	4	28	4	28	10	22
3	11	62	11	62	13	60
4	40	33	41	32	55	18
5	15	57	15	57	33	39
6	29	53	29	53	56	26
7	8	45	8	45	13	40
8	13	29	13	29	22	20

^a^FRA: females with reproductive activities.

^b^EPPGI: Extraction Protocol of Pregnancy and Gestation Information.

**Table 7:** FRA^a^ recognition results for different privacy methods in different years (1 being “there is maternity information” and 0 being “no maternity information”).

Year	Quality inspection		EPPGI^b^		Diagnosis history and marital history	
	0	1	0	1	0	1
						
2010	14	9	14	9	19	4
2011	18	10	18	10	23	5
2012	19	18	19	18	29	8
2013	42	40	42	40	55	27
2014	46	59	46	59	60	45
2015	33	75	34	74	55	53
2016	42	93	42	93	69	66
2017	63	130	64	129	95	98
2018	60	107	60	107	86	81
2019	27	58	27	58	36	49
2020	13	24	13	24	16	21

^a^FRA: females with reproductive activities.

^b^EPPGI: Extraction Protocol of Pregnancy and Gestation Information.

### Privacy Protection Strategies

The privacy-protection strategies were developed based on the above results. Given that we used regular expressions to identify SRI, additional text surrounding the recognized maternity information needs to be concealed to prevent privacy exposure through context. We randomly selected 1000 EMRs of pregnancy patients for static data desensitization to create a masked sample of EMRs. A total of 2 independent reviewers (Aixin Guo and Wenna Liu) were assigned to manually extract any form of pregnancy and gestation information from the masked samples. Furthermore, the risk of unnecessary privacy exposure was stratified by the frequency of recognition. The text of medical records, having the highest recognition frequency, should be handled with the utmost caution. In contrast, test and examination records are less frequently identified with SRI. It is important to note that the frequency of recognition does not fully represent the risk of privacy leakage, which will be further analyzed in the discussion section.

## Discussion

### Principal Findings

#### Overview

This study is one of the first large-scale investigations into privacy leakage and FRA identification of Chinese EMRs, focusing on the frequency of recognition. The originality of this work can be summarized in 3 key aspects.

#### Originality in Exploring New Observations

The accessibility of EMR inevitably leads to uneven privacy protection awareness among different EMR users. The importance of reliable privacy protection methods has been extensively discussed in the literature, emphasizing their critical role in the successful implementation of EMRs in health care institutions [18,19]. Sensitive information regarding pregnancy, gestation, and abortion is routinely included in EMRs, raising concerns about unnecessary exposure [20,21]. In 2021, the Personal Information Protection Law of the People’s Republic of China came into effect, which clarified the rights and responsibilities related to the use of personal privacy information [22]. However, prior to this study, there has been little to no effort to address the highest standards of patient privacy protection protocols during RWE studies. To the best of our knowledge, this is the first study in China to use a national-level EMR database to quantitatively evaluate the exposure risk of privacy information related to women’s reproductive health. This study aims to enhance protection strategies in this area.

#### Originality in Designing New Experiments

The attributes and structure of Chinese EMRs are unique in terms of terminology and data standards. Accurate and comprehensive recognition of maternity information is widely reported to play a critical role in effective privacy protection and the evaluation of RWE [23,24]. While researchers have been working to improve the accuracy of SRI identification in non-Chinese EMRs [25,26], to the best of our knowledge, no prior research has focused on the accurate and complete extraction of FRA from Chinese EMRs. Traditional diagnosis-based patient extraction protocols typically use diagnosis codes, such as the International Classification of Diseases, which have 2 major limitations.

First, the records in the diagnosis sheet are often incomplete. Due to inconsistencies in Chinese EMR documentation, physicians do not always record pregnancy and gestation information as a diagnosis, especially when the patient’s primary complaint is unrelated to maternity. This leads to lower recall rates and potential recall bias. Second, due to the complexity and inconsistency of coding systems in Chinese EMRs, using codes for patient identification is more complicated than using regex, and it is nearly impossible to list all encodings exhaustively. Furthermore, regex can be widely adopted across different sub–data sets of Chinese EMRs. Although the diagnostic sheet contains the major SRI, most Chinese EMRs are still stored in text format without code mapping.

Compared to traditional diagnosis-based patient inclusion methods, the EPPGI method provides more precise results in a practical manner. Whether patient- or visit-based records, EPPGI extracts significantly more FRA with a high precision rate.

#### Originality in Contributing New Knowledge

Our results demonstrate that traditional fixed-site data masking procedures lead to considerable unnecessary exposure of privacy information. For instance, patients’ HCG test results or delivery procedures are commonly recorded in sub–data sets that cannot simply be concealed during RWE studies and clinical use. The combination of pregnancy and gestation information can even infer the incidence of abortion, which is highly confidential in China. Accurate and complete recognition of maternity information is essential for flawless privacy protection.

EPPGI method first identified pregnancy and gestation information across entire Chinese EMRs. For the identified information, it is practical and convenient to use data desensitization techniques, including data invalidation, data offset, and symmetric encryption, to prevent the misuse of private data. Based on the EMRs in CRDS, we determined the optimal length of additional concealed text to retain most medical information. Additionally, the quantified recognition frequency of pregnancy and gestation information helps researchers use EMRs wisely to avoid unnecessary privacy leakage. Although the frequency of identification cannot fully determine the risk of privacy leakage, which is also associated with the complexities of data desensitization, these results highlight the richness of private information ingrained in EMRs.

From a data asset management perspective, quantifying the risk of privacy leakage is critical under the strict Personal Information Protection Law. Based on statistical results and actual data mining practices, SRI is widely stored in Chinese EMRs, requiring data desensitization when using any EMR sub–data sets. For test results and structured data, the difficulty of data desensitization is relatively lower than that for plain text medical records, given the explicit nature of sensitive data entities and the low probability of reinference from context. Similar to the hazard classification of chemicals, health care data users should be aware of the richness of private information and the risk of unnecessary privacy exposure in EMRs. Maternity information is considered one of the most sensitive types of privacy for women, and our results provide a crucial reference for data users to assess related risks in Chinese EMRs for the first time.

### Limitations

Overall, this work provides justification for assessing privacy leakage risk and offers a reference for effective privacy protection in Chinese EMRs. However, the proposed study has several limitations. First, the frequency of sensitive information and the privacy risk estimated in our case study are primarily based on the EMRs of a renal disease database. While there are official directions and guidelines for composing EMRs in China [9], discrepancies exist between the CRDS and other data networks in terms of data structure and operating environment. Specific protocols and variables should be optimized for generalizations.

Furthermore, the study is limited by its data scale, covering only 688,268 FRA in the CRDS. This limited scope suggests the need for further research involving larger data sets to validate and refine our findings.

### Conclusions

Finding an effective and practical way to protect private information in EMRs is both meaningful and useful. We have demonstrated the feasibility of applying the EPPGI method to EMRs from 19 hospitals in different regions. We believe that EPPGI can provide a valuable reference for patient inclusion in any maternity-related studies using Chinese EMRs. Our protocols, designed for Chinese EMR systems, enable the accurate and complete recognition and extraction of pregnancy and gestation data, ensuring its effective protection. Compared to traditional methods of FRA inclusion, the EPPGI method provides more comprehensive results.

## Appendix

Multimedia Appendix 1 The detailed rules and regex of EPPGI (Extraction Protocol of Pregnancy and Gestation Information).

Multimedia Appendix 2 Source code.

## Abbreviations

CRDS: Chinese Renal Disease Data System
EMR: electronic medical record
EPPGI: Extraction Protocol of Pregnancy and Gestation Information
FRA: females with reproductive activities
HCG: human chorionic gonadotropin
RWE: real-world evidence
SRI: sensitive reproductive information
STROBE: Strengthening the Reporting of Observational Studies in Epidemiology

## Data Availability

The data sets analyzed during this study are not publicly available due to the sensitivity of hospitals’ data but are available from the corresponding author upon reasonable request. The source code is available in Multimedia Appendix 2.
