# Supplementary material for: Effective Privacy Protection Strategies for Pregnancy and Gestation Information From Electronic Medical Records: Retrospective Study in a National Health Care Data Network in China
Source: J Med Internet Res. 2024 Aug 20;26:e46455. doi: 10.2196/46455 (PMC11372317; doi:10.2196/46455)
Supplement: Multimedia Appendix 1 [file jmir_v26i1e46455_app1.docx]

1. Doctor’s order – drug:

Regex (use R as example):

[（‘麦角生物碱|催产素|麦角新碱|地诺前列素|地诺前列酮|麦角生物碱 | 催产素 | 麦角新碱 | 地诺前列素 | 地诺前列酮 | 吉美前列素 | 卡前列甲酯 | 硫前列酮 | 米索前列醇 | 米菲司酮’)]

Note: Taking these as keywords, these drugs are mainly used for oxytocin, and are most commonly used for miscarriage, but other uses are not excluded. Preliminary consideration can be given to the situation of pregnancy and childbirth, combined with other information to make a diagnosis.

1. Diagnosis:

Diagnosis Code:

O00|O08.006|O08.104|O08.105|O08.106|O08.302|O08.806|O36.7|O83.3|O10|O11|O12|O13|O14|O15|O16|O20|O21|O22|O23|O24|O25|O26|O28|O29|O30|O31|O33|O34|O35|O36|O40|O41|O42.2|O43|O44|O45|O46|O47|O48|O63|O98|O99|Z32.1|Z33|Z34|Z35|Z64.0

Note: Since there are considerable complexity and diversification in codes of pregnancy-related diseases, diagnosis code were not used.

Regex:

[(‘怀孕|孕\\d\\d?产[\\d\\d?|孕.{0,5}周|早孕|晚孕|中孕|新生儿|顺产|流产|待产|助产|难产|平产|早产|死产|死胎|引产|剖腹产|剖宫产|刮宫术|足月产|妊娠|宫外孕|分娩|人流|药.?流|胎心|胎盘|头盆|先露|子痫|胎死|羊水|胎动|胎儿|胎膜|胎心|胎体|胎位|胚胎[^性|型]|活胎|脐带|胚芽|脐动脉|妊高症|妊高征|产前|产钳|产后|产褥|清宫|唐氏筛选|G\\d\\d?P\\d+|胎盆不称|单胎|双胎|多胎|\\d胎|[一|二|三|四|五|六|七]胎|人工受精](about:blank)’)]

remove at the same time:

[(‘胚胎.?(癌|腺瘤|(横纹肌)?肉瘤|囊肿|发育不良性神经上皮.?肿瘤|源性或神经源性肿瘤)|胎儿上皮型’)]

1. Test:

The result of test item HCG (human chorionic gonadotropin) is:

hcg>=10ng/ml, or hcg>=25IU/L, or a positive pregnancy test

remove at the same time: 葡萄胎 | 侵蚀性葡萄胎 | 绒毛膜癌 | 绒毛膜腺瘤

Note: Patients with common gestational trophoblastic diseases (mole, invasive mole, choriocarcinoma, chorioadenoma) that cause elevated hcg should be removed.

1. Exam:

Match regex of the check result description or doctor's diagnosis opinion (check_result, check_doctor_desc) field in the check report text. The rules are the same as the diagnosis.

1. Medical records:

The rule of regex matching is same as diagnosis

1. Structured admission records:

The rule of regex matching in histories of diseases, tobacco and alcohol, surgery, and toxic exposure are the same as the diagnosis.

Chief complaint: Since most of the chief complaint of menopause is related to pregnancy, the keyword 'menorrhea (停经)' is added in the regex rules and the rest are the same as the diagnosis.

Family history: According to the structured format model: whether there is a disease history (whether)+relationship (relation)+disease name (diag)

[!weather %like% ‘否认|无' & (RELATION=='' | RELATION=='')] + the regularity condition for disease names(diag) are the same as regex rules in diagnosis.

Note: some personal SRI could be recorded in family history as well.

Kinship relationships other than child relationships, such as parents, etc. are excluded.

Marriage history: according to the structured model:

[生育状况==是|怀孕次数>0|生产次数>0||流产次数>0|死产次数>0|子数量>0|女数量>0|子女数量>0]
